# Spitz Melanocytic Tumors: A Fascinating 75-Year Journey

**DOI:** 10.3390/genes15020195

**Published:** 2024-01-31

**Authors:** Kyriakos Chatzopoulos, Antonia Syrnioti, Konstantinos Linos

**Affiliations:** 1 Department of Pathology, Aristotle University, 54636 Thessaloniki, Greece; kchatzop@auth.gr (K.C.); asyrnioti@auth.gr (A.S.); 2 Department of Pathology and Laboratory Medicine, Memorial Sloan Kettering Cancer Center, 1275 York Avenue, New York, NY 10065, USA

**Keywords:** spitz, spitzoid, melanocytic, atypical spitz tumor, melanoma, nevus

## Abstract

Over the last 75 years, our understanding of Spitz lesions has undergone substantial evolution. Initially considered a specific type of melanoma, the perception has shifted towards recognizing Spitz lesions as a spectrum comprising Spitz nevi, Spitz melanocytomas, and Spitz melanomas. Spitz lesions are known for posing a significant diagnostic challenge regarding the distinction between benign neoplasms displaying atypical traits and melanomas. A comprehensive understanding of their molecular basis and genomic aberrations has significantly improved precision in classifying and diagnosing these challenging lesions. The primary aim of this review is to encapsulate the current understanding of the molecular pathogenesis and distinct clinicopathologic characteristics defining this intriguing set of tumors.

## 1. Introduction and Historical Perspective

It has been 75 years since the hallmark publication of “Melanomas of childhood” by Sophie Spitz in the *American Journal of Pathology* [1]. Therein, Spitz, who was an assistant professor of pathology at the Sloan-Kettering division of Cornell University in New York at the time [2], presented a case series consisting of 13 pediatric lesions initially diagnosed as melanoma. Although 12 of the 13 patients followed an indolent clinical course, a 12-year-old girl eventually died of metastatic disease [1]. 

It is now apparent that most of these lesions represented what is currently recognized as Spitz nevus. Sophie Spitz, though, held the belief that these lesions represented true malignancies that happened to have a less aggressive clinical course, potentially influenced by hormonal factors [1]. This belief was reinforced by the case of the 12-year-old girl included in the initial series who unfortunately succumbed to metastatic disease [1]. A few years later, in 1953, Sophie Spitz and her husband Allen published a clinicopathologic study of melanomas. In this study, they extended their observations on what were initially termed “melanomas of childhood” by demonstrating that these melanomas can occasionally arise in adults. They identified a rare subset of them that had metastatic potential, particularly those that displayed prominent cellular pleomorphism and a “cordlike” invasion pattern [3]. 

Sophie Spitz was not only a brilliant pathologist but also a prominent advocate of the Pap smear at a time when most of the scientific community had been skeptical about that particular method [4]. Additionally, she was among the first investigators to describe the carcinogenic potential of aromatic amines [5]. It is profoundly unfortunate that such a prominent figure in early cancer research eventually succumbed to metastatic colon cancer in 1956, at the age of 46 [2]. Following her passing, a multitude of studies have been undertaken with a primary focus on comprehending the clinicopathologic and etiopathogenic aspects of this enigmatic entity she dedicated herself to. 

In 1960, Kernen and Ackerman published a series of 27 cases referred to as “juvenile melanomas”. In their study, they contended that tumors with metastases were in fact misdiagnosed genuine melanomas which displayed a distinctive spindle cell or epithelioid morphology [6]. 

A few years later, in 1967, the term “Spitz nevus” made its debut in the scientific literature. It was proposed by the Queensland Melanoma Project Committee in Australia as a tribute to the late Sophie Spitz [7]. During the 1970s, there were descriptions of several Spitz nevus (SN) variants, including the pigmented spindle cell nevus as well as the desmoplastic, pseudogranulomatous, and nevus with halo reaction variants [8]. Notably, in 1979 Kamino and her colleagues introduced the concept of PAS-positive, diastase-resistant, pink globules that are frequently observed in SN. These globules have since been referred to as Kamino bodies [9]. 

Since the 1970s, it has become evident that some spitzoid lesions pose a considerable challenge when it comes to classification, making it difficult to determine whether they should be labeled as nevi or melanomas. In 1969, the term “Atypical Spitz Tumor” was introduced in the literature [10]. This term was coined to describe spitzoid tumors displaying atypical features such as increased cellularity, asymmetry, decreased maturation, deep extension, and cytologic atypia. Despite these atypical features, these lesions did not entirely meet the morphologic criteria to be definitively categorized as melanomas [11]. 

In the 2010s, the advent of next generation sequencing (NGS) provided new insights into the pathogenesis of Spitz lesions. Studies utilizing NGS technology have shed light on the molecular underpinnings of these lesions, revealing the frequent presence of oncogenic fusions as a hallmark molecular event. This discovery has significant diagnostic implications and potentially opens the door to therapeutic possibilities [8].

From a clinical perspective, Spitz tumors represent a spectrum of lesions, with common Spitz nevus on the one end, frequently arising on the face or extremities of children; they are well circumscribed and symmetrical [12] and show bland histologic features [13]. Although most Spitz nevi are pigmented, nonpigmented lesions are more frequently seen in the head and neck area [14]. Spitz melanoma represents the other end of the spectrum, encompassing tumors that are usually overt malignancies by histology, although the distinction between Spitz melanomas and benign nevi can be problematic solely based on histology, particularly in older patients [15]. As will be discussed in detail below, the molecular background of Spitz lesions is quite complex and often includes interactions between genetic and hormonal factors [16].

The purpose of the present review is to summarize the existing knowledge regarding the molecular pathogenesis and distinctive characteristics of this fascinating group of tumors.

## 2. The Clinicopathologic Spectrum of Spitz Lesions

### 2.1. Spitz Nevus

Clinically, Spitz nevi (SN) primarily occur in individuals below the age of 20 and show a predilection for the extremities [17,18]. Typically, they manifest as a circumscribed, smooth-surfaced plaque, papule, or nodule, with a diameter of less than 6 mm, and exhibit a distinctive pink or pink-red color. 

Microscopically, SN are characterized by a well-defined, symmetrical, and often wedge-shaped proliferation of large, spindle-shaped, or epithelioid cells arranged in vertically oriented nests at the dermo-epidermal junction [19,20]. This proliferation frequently extends into the superficial dermis, demonstrating maturation. Notably, SN typically display zonation or uniformity from one side of the lesion to the other. They are low in cellularity, lack solid growth or confluence of nests, and often exhibit retraction clefts between the nests and the epidermis. Superficial, regular deposition of melanin is another frequent feature, and the overlying epidermis is typically hyperplastic without ulceration or effacement. Pagetoid spread in SN, if present, is usually focal and confined to the center of the lesion and the lower half of the epidermis. Cytological atypia is mild, maintaining a preserved nucleus-to-cytoplasmic ratio. Mitotic activity is low, with fewer than two mitotic figures per mm^2^, and an absence of atypical or deep mitoses. Kamino bodies may also be observed, while solar elastosis is typically absent [19,20]. Dermal inflammation, although common, only occasionally results in what manifests clinically as halo nevus [17]. 

Various histopathologic variants of SN have been described, including angiomatoid, desmoplastic, epithelioid, pagetoid, pigmented spindle cell nevus, plexiform, and spindle cell [21]. As will be discussed in detail below, certain histopathologic variants are more likely to be reflective of specific underlying molecular alterations. 

SN behave in a benign clinical manner, with patients remaining free of recurrence after complete surgical excision [22]. 

### 2.2. Spitz Melanocytoma (Atypical Spitz Tumor)

Spitz melanocytomas can occur at any location and age, but they most frequently affect individuals between the ages of 10 and 40 [18]. They typically present as hypopigmented or variegated-in-color plaques or nodules, often larger than conventional SN, with a diameter ranging from 7 to 10 mm and an irregular surface. 

Microscopically, Spitz melanocytomas may exhibit asymmetry and lack zonation, and they frequently display a confluence of irregular, highly cellular nests or nodules of large, spindle-shaped, or epithelioid cells. Involvement of the deeper dermis or subcutis, accompanied by partial or absent maturation, is often noted. Clefting between the melanocytes is typically irregular and occurs in subepidermal regions. Superficial, regular melanin deposits might also be observed. The overlying epidermis may be ulcerated, often exhibiting complete effacement or consumption. Pagetoid scatter is more extensive than in conventional SN and may be peripheral or involve the upper half of the epidermis. Melanocytes in Spitz melanocytomas typically exhibit moderate or marked atypia, characterized by an increased nucleus-to-cytoplasmic ratio and nuclear hyperchromasia [19,20]. The mitotic rate ranges from 2 to 6 figures per mm^2^ [18], and notably, most cases of Spitz melanocytomas lack atypical mitotic figures. In contrast to SN, Spitz melanocytomas may exhibit deep mitoses. In some cases, necrosis may also be identified. Kamino bodies are generally few or absent, while solar elastosis is uncommon [19]. 

Despite these worrisome features, Spitz melanocytomas typically follow an indolent clinical course with minimal lethal potential [22].

### 2.3. Spitz Melanoma

Finally, malignant Spitz tumors or Spitz melanomas (SMs) can also occur at any location and age but are most common among individuals over the age of 40. They usually present as a changing or enlarging poorly circumscribed plaque or nodule, with a diameter of more than 10 mm, an irregular surface, and color variegation. 

On microscopic examination, similarly to Spitz melanocytomas, Spitz melanomas are asymmetrical, lack zonation, and exhibit a confluence of irregular, highly cellular nests or nodules of large, spindle-shaped, or epithelioid cells. These typically extend into the deeper dermis or subcutis, without any maturation. Irregular, subepidermal clefting between melanocytes may be noted. Spitz melanomas classically show complete effacement of the epidermis, which may also be ulcerated. Pagetoid scatter is also extensive, frequently peripheral, and may involve the upper half of the epidermis. Cellular atypia is once again moderate or marked, with an increased nucleus-to-cytoplasmic ratio and nuclear hyperchromatism or prominent eosinophilic nuclei. Atypical and deep mitotic figures are easily found, commonly more than 6 mitotic figures per mm^2^ [18,19,20]. Another distinctive characteristic is the presence of irregular melanin deposits, especially in deeper sites of the lesion. Solar elastosis is also frequently observed in Spitz melanomas, while Kamino bodies are absent. In keeping with their malignant nature, SMs can develop disseminated disease [22].

As will be discussed below, the morphologic spectrum from totally benign to overtly malignant lesions probably reflects the molecular spectrum of alterations that occur during tumorigenesis [23]. Therefore, rendering a dichotomous diagnosis of a benign versus malignant Spitz tumor can be both challenging and problematic to reproduce among different diagnosticians [23]. Although detailed morphologic examination may occasionally reveal helpful findings, the existence of morphologic variants of Spitz lesions, such as pseudogranulomatous [24] or desmoplastic SN [25], can further complicate the differential diagnosis. In this context, it is not uncommon that the final diagnosis must be deferred to the integration of molecular findings into the morphological assessment, a process that can significantly increase interobserver agreement between different pathologists [26]. Therefore, a clear understanding of the molecular alterations encountered in Spitz lesions by pathologists is a *sine qua non*.

### 2.4. BRAF-Mutated and Morphologically Spitzoid (BAMS) Nevi and Tumors

The increased usage of BRAF V600E immunohistochemistry led to the recognition of a subset of benign or intermediate-grade melanocytic lesions with Spitzoid features driven by a canonical *BRAF* mutation [27]. These are pigmented lesions that arise in the extremities and show mixed epithelioid and spindled features and moderate atypia, usually without Kamino bodies [27]. Occasionally, BAMS can involve a sentinel lymph node, although distant metastatic spread has not been reported [27]. Although these enigmatic lesions show morphologic features seen in the spectrum of Spitz melanocytoma [27], they probably do not represent genuine Spitz tumors, but rather their morphologic mimickers [28].

## 3. Molecular Pathology of Spitz Lesions

### 3.1. Early Findings including HRAS Alterations

During the early stages of molecular research on nevi, it became apparent that MAPK pathway activation results from *BRAF* mutations in common nevi and small congenital nevi [29], *NRAS* mutations in medium-sized congenital nevi [29], *GNAQ* or *GNA11* mutations in blue nevi [30], and *HRAS* mutations in a subset of Spitz nevi [31]. Spitz melanomas often exhibit more mutations than Spitz melanocytomas and Spitz nevi, resulting in a higher tumor mutational burden (TMB), occasionally including a UV mutational signature [32]. 

**Figure 1:**
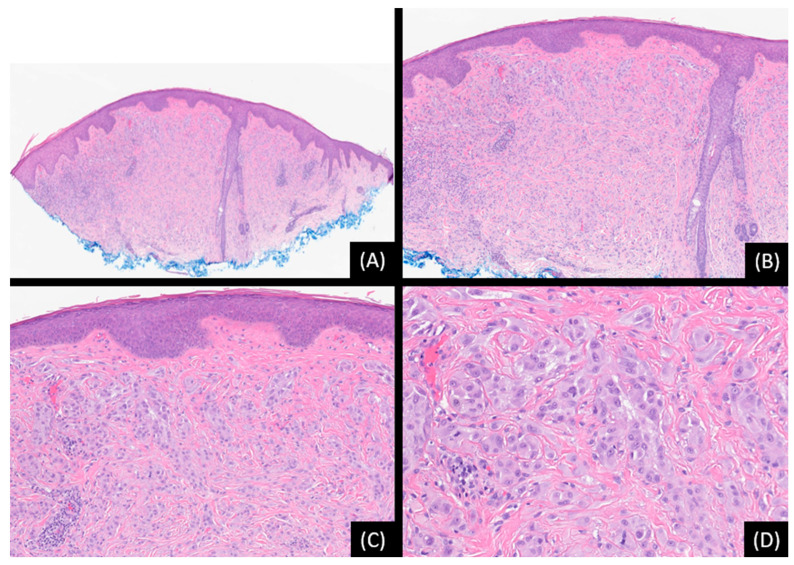
Spitz nevus with 11p gain. (**A**,**B**): Low and medium-power view depicting a dome-shaped lesion with deep growth into the dermis (hematoxylin and eosin stain [H&E], original magnification 20×, 50×). (**C**): There is mild fibrosis among tumor cells (H&E, original magnification 100×). (**D**). The cells are mostly epithelioid, with eosinophilic cytoplasm, round, or ovoid nuclei with minimal pleomorphism, and are grown as single units or in nests (H&E, original magnification 200×). Single Nucleotide Analysis (SNP) array analysis detected 11p chromosomal gain.

Understanding the unique molecular features of SN started in the late 1990s with the observation in 1999 that some SN have 11p chromosomal gains and lack the frequent chromosomal deletions seen in melanomas, making them genetically distinct [33]. Following this initial observation, it quickly became apparent that these tumors also frequently harbor *HRAS* oncogenic mutations and display distinctive histopathologic features, including a larger size, desmoplasia, and deeper infiltrative growth [34] (Figure 1). These features were confirmed from subsequent studies, including a case series of 24 *HRAS*-mutant Spitz tumors published in 2010 [35]. Of note, recent research has shown that 11p chromosomal gains are not solely confined to desmoplastic Spitz nevi and can also be observed in non-desmoplastic and papillomatous lesions, as well as atypical melanocytic tumors exhibiting deep bulbous growth [36]. The frequent presence of multiple copy number variations (CNVs) in melanomas, in contrast to the occasional 11p gains in SN, allowed for the use of comparative genomic hybridization (CGH)/single nucleotide polymorphism (SNP) array analysis [37] and later fluorescent in situ hybridization (FISH) for distinguishing difficult-to-classify SN from melanoma [38,39,40]. 

It appears that the acquisition of *HRAS*-activating mutations frequently represents one of the hallmark events that can trigger the development of agminated SN, characterized by the emergence of multiple, sometimes hundreds, of morphologically and molecularly identical lesions throughout the body, a clinically concerning situation that may arise from the transformation of a pre-existing nevus spilus [41]. However, case reports of patients with agminated SN lacking *HRAS* mutations and instead harboring fusions including *PRKCA* or *ROS1* have been published [42,43], including a 30-month-old girl with a recurrent agminated SN of the face, which had a *GOPC::ROS1* fusion and responded clinically to treatment with crizotinib [44].

### 3.2. Oncogenic Fusions

In 2014, Wiesner et al. published a study of 140 Spitz neoplasms, including Spitz nevi, Spitz melanocytomas, and Spitz melanomas. They identified gene fusions including *ROS1*, *NTRK1*, *ALK,* and less frequently *BRAF* or *RET*, detectable in approximately half of SN and Spitz melanocytomas and almost 40% of SMs [45]. In general, testing by next-generation sequencing (NGS) has allowed for the detection of characteristic oncogenic fusions, expanding the morphologic spectrum of spitzoid lesions to older patients whose tumors might have not been diagnosed as overt Spitz tumors without the means of molecular investigation [46]. 

In later studies, it was demonstrated that up to 80% of SN have oncogenic fusions including a gene encoding a receptor tyrosine kinase such as *ALK*, *FGFR1*, *MET*, *MERTK*, *NTRK1/2/3*, *RET,* or *ROS1* or a gene encoding a serine-threonine kinase, such as *BRAF*, *ERBB4*, *MAPK3K3*, *MAP3K8*, or *PRKDC*. Moreover, these fusions are mutually exclusive, and the fusion transcript is highly expressed in the majority of cases. These fusions result in a constitutively activated kinase domain, promoting cellular growth and proliferation and acting as an oncogenic driver [47]. It is interesting that certain fusions tend to be correlated with specific phenotypes, an observation that can be helpful in triaging tumors for further molecular testing in clinical practice.

**Figure 2:**
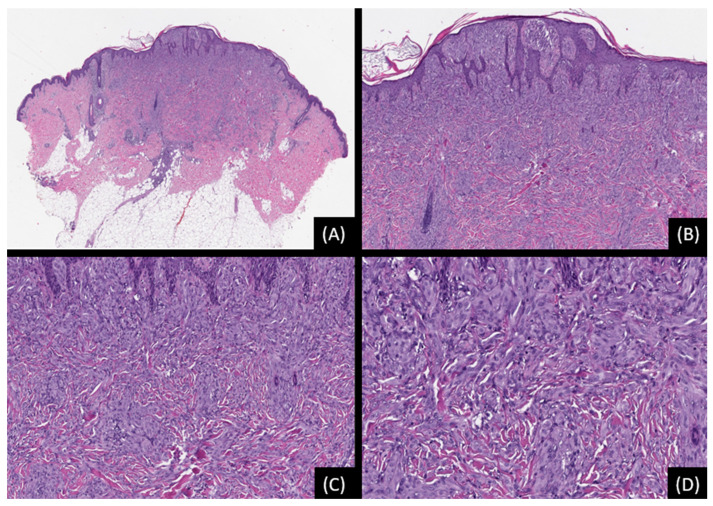
Spitz nevus with *TMP3::ALK* fusion. (**A**): Low-power view showing a relatively large, poorly demarcated melanocytic proliferation extending into the deep dermis and superficial subcutis (H&E, original magnification 20×). (**B**,**C**): The lesion is characterized by a confluence of irregular, highly cellular nests or nodules of large epithelioid and spindle-shaped cells. Focal effacement of the overlying epidermis is also observed (H&E, original magnifications 50× and 100×, respectively). (**D**): Mild to moderate nuclear pleomorphism is appreciated (H&E, original magnification 200×). SNP array analysis did not reveal any unbalanced genomic aberrations.

**Figure 3:**
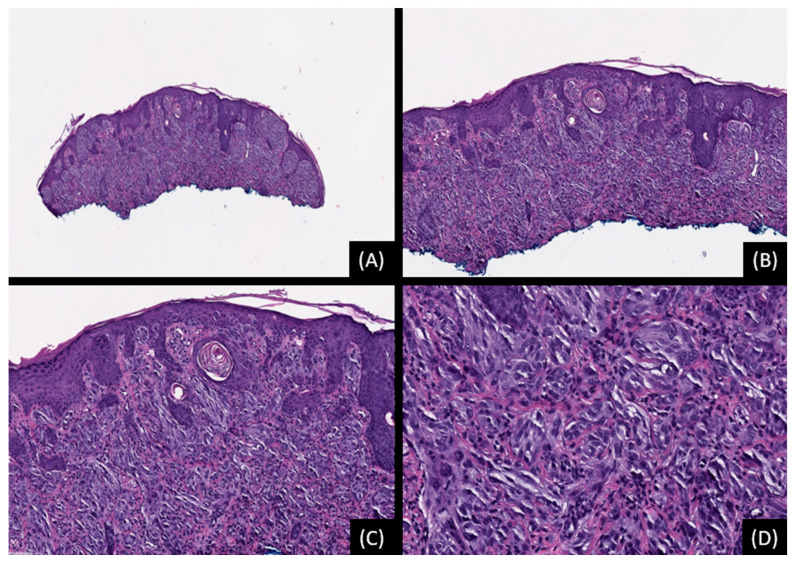
Spitz melanocytoma (Atypical Spitz Tumor) with *TMP3::ALK* fusion. (**A**): Low-power magnification showing a highly cellular spindle and epithelioid melanocytic proliferation (hematoxylin and eosin stain [H&E], original magnification 20×). (**B**,**C**): Confluent irregular nests and nodules of epithelioid and spindle-shaped cells are appreciated (H&E, original magnifications 50× and 100×, respectively). (**D**): The tumor cells exhibit voluminous eosinophilic to basophilic cytoplasm with nucleomegaly and moderate cytologic atypia. (H&E, original magnification 200×). SNP array analysis detected low level loss of 2p and gain of 2q, low level gain of chromosome 4, low level loss of chromosome 9, low level gains in segments 15q11.2-q21.3 and 15q23-qter, low level loss of 17q, and low level gain of chromosome 22. The tumor was negative for *TERT* promoter mutations. The findings indicate genetic instability, and despite being severely atypical, the overall morphologic and cytogenetic data are not diagnostic of Spitz melanoma.

Multiple studies have reported histopathologic findings distinctive of *ALK*-fused Spitz tumors, which represent between 8–26% of all Spitz lesions [8]. They tend to be larger in size, with deep dermal extension, a fascicular or plexiform growth pattern, and nests of large, spindle-shaped cells [48,49,50,51] (Figure 2 and Figure 3). They also tend to be relatively amelanotic and have a paucity of epithelioid cells [48]. Common fusion partners for *ALK* include *TPM3* and *DCTN1* [52], although less common fusion partners have also been described, including an interesting case of metastatic pediatric SM with *ALK* fusion leading to promoter hijacking (C2orf42::*ALK*) [53]. Although *ALK*-fused Spitz tumors can be detected by immunohistochemistry [48], the selection of the suitable antibody clone may be important, as illustrated by a Spitz melanocytoma with *MLKPH::ALK* fusion which was non-reactive with the ALK1 antibody clone but showed reactivity with the 5A4 antibody [54]. This has also been reported in neoplasms of other lineages, most notably *ALK*-rearranged non-small-cell lung cancer (NSCLC) [55,56].

Up to 20% of Spitz tumors are *NTRK*-fused [8], with *NTRK1* being the most common of the three *NTRK1/2/3* genes to be involved [57]. *NTRK1*-fused Spitz tumors only infrequently show the fascicular growth of fusiform cells of their *ALK*-fused counterparts [48]. Instead, they are more likely to display Kamino bodies and they typically consist of smaller spindle cells in a nested architectural pattern occasionally containing rosette-like structures [49,58]. In addition, *NTRK1*-fused tumors show elongated and branching rete ridges [58]. Although a pseudo-schwannoma architectural pattern has been suggested as a characteristic feature of *NTRK3*-fused tumors [50], it seems that *NTRK2/3*-fused tumors are morphologically indistinguishable from their *NTRK* wild-type counterparts most of the time [59,60]. Common fusion partners include *LMNA* and *TP53* for *NTRK1* [8], *SQSTM1*, *TRAF2,* and *TFG* for *NTRK2* [61], and *ETV6*, *MYH9,* and *MYO5A* for *NTRK3* [60].

**Figure 4:**
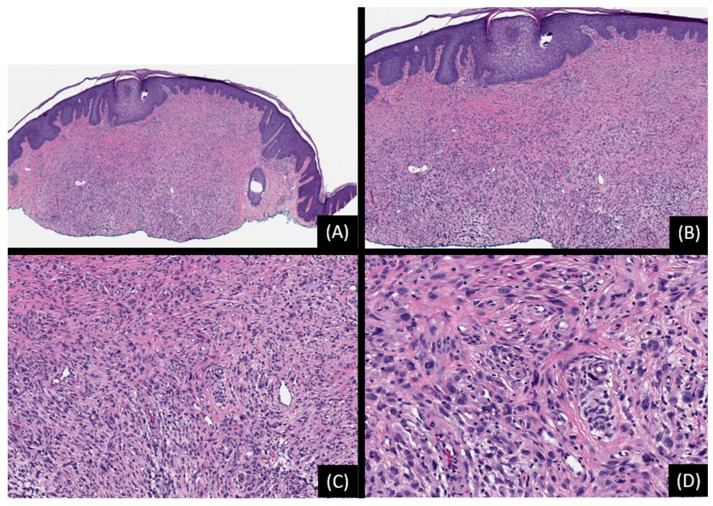
Spitz melanocytoma (Atypical Spitz Tumor) with *AKAP9::BRAF* fusion. (**A**,**B**): Low-power magnification showing a dome-shaped, intradermal cellular melanocytic proliferation (hematoxylin and eosin stain [H&E], original magnification 20× and 50×, respectively). (**C**,**D**): The neoplasm consists of spindle and epithelioid cells growing in nests and short fascicles. The tumor cells have eosinophilic cytoplasm and show moderate nuclear pleomorphism (H&E, original magnification 100× and 200×, respectively). The tumor showed no unbalanced genomic genomic aberrations by single nucleotide polymorphism (SNP) array. Testing by FISH was negative for homozygous deletion of *CDKN2A* (p16, 9p21). The findings are atypical but not diagnostic of melanoma.

**Figure 5:**
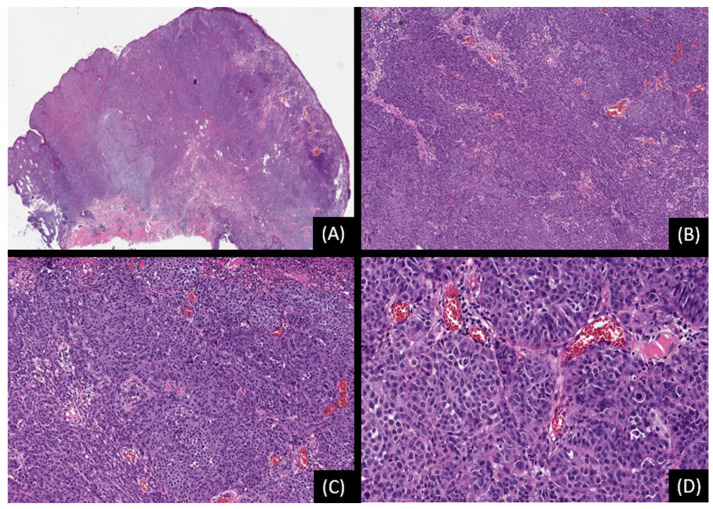
Melanoma with *AGK::BRAF* fusion. (**A**): Low-power magnification showing a cellular neoplasm with deep invasive growth into the dermis (hematoxylin and eosin stain [H&E], original magnification 10×). (**B**,**C**): The tumor is hypercellular growing in sheets (H&E, original magnification 50× and 100×, respectively). (**D**): Tumor cells have eosinophilic to basophilic cytoplasm and hyperchromatic nuclei with increased nuclear-to-cytoplasm ratio and moderate nuclear pleomorphism (H&E, original magnification 200×).

**Figure 6:**
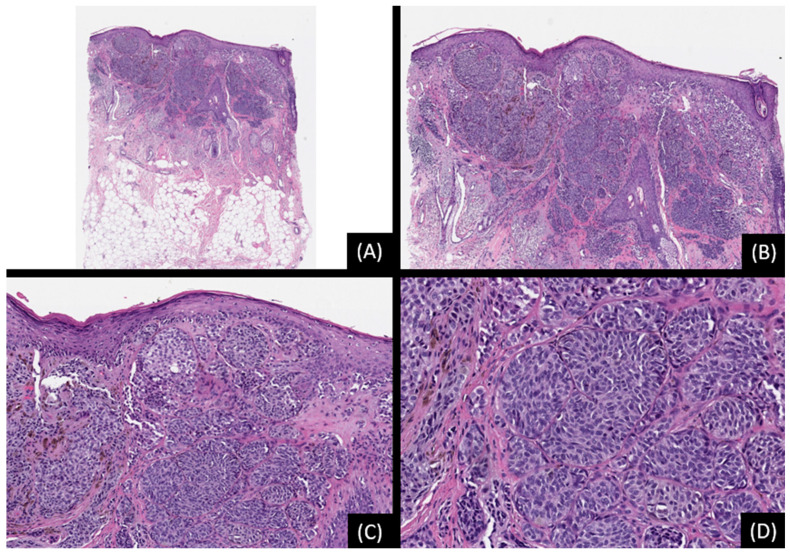
Melanoma with *CMAS::BRAF* fusion. (**A**): Low-power magnification showing an asymmetrical, cellular neoplasm with infiltrative/invasive growth into the dermis (hematoxylin and eosin stain [H&E], original magnification 20×). (**B**–**D**): The tumor grows predominantly in nests and shows mostly epithelioid cytomorphology, with eosinophilic cytoplasm and hyperchromatic nuclei with atypia. Melanin pigment is also evident (H&E, original magnifications 40×, 100× and 200×, respectively).

*BRAF* fusions can be detected in approximately five percent of Spitz tumors. They typically show epithelioid morphology, less in a nested and more in a sheet-like architectural pattern [49,50], with cellular atypia and desmoplasia more often seen in Spitz melanocytomas [49,62] (Figure 4, Figure 5 and Figure 6). Among different fusion partners, *CLIP2* is the most frequent, with other partners including *SKAP2*, *AGK*, *MYO5A*, *MLANA* [62], *NRF1, SOX6*, *EMLR4,* and *BAIAP2L1* [8]. The presence of concurrent mutations is not an uncommon finding. *BRAF*-fused tumors typically follow an indolent clinical course [62], although a subset may progress to melanoma [49].

**Figure 7:**
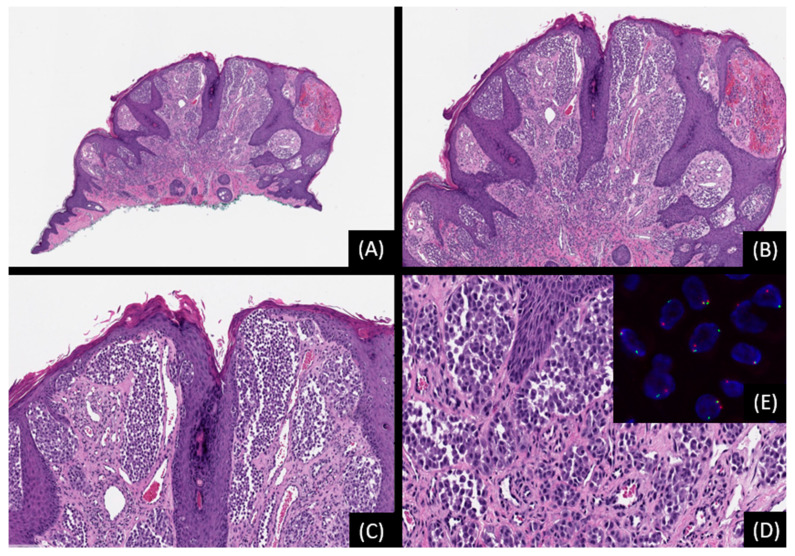
Spitz nevus with *TMP3::ROS1* fusion. (**A**): Low-power magnification shows a dome-shaped, compound melanocytic proliferation associated with epidermal hyperplasia (hematoxylin and eosin stain [H&E], original magnification 20×). (**B**,**C**): The lesion is composed of predominantly epithelioid cells arranged in nests of various sizes. (H&E, original magnifications 50× and 100×, respectively). (**D**): Epithelioid tumor cells with hyperchromatic nuclei, increased nuclear-to-cytoplasmic ration, and moderate cellular atypia (H&E, original magnification 200×). (**E**): Break-apart FISH reveals separation of red and green signals indicative of *ROS1* rearrangement. SNP array analysis did not reveal any unbalanced genomic aberrations.

**Figure 8:**
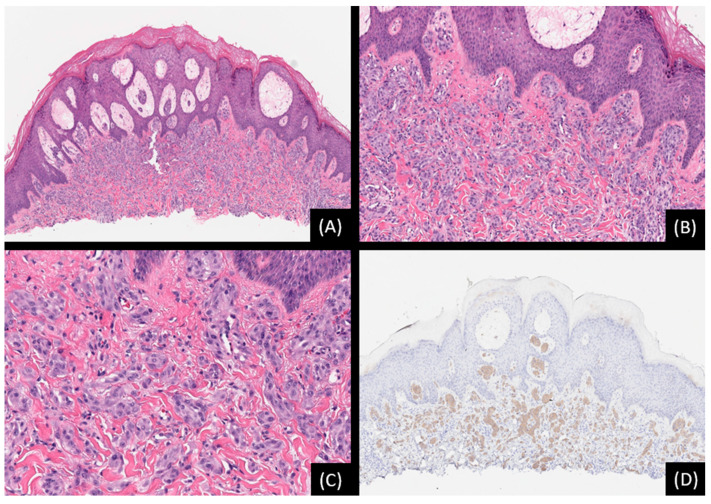
Spitz nevus with *PPWWP2A::ROS1* fusion. (**A**): Low-power view depicting a predominantly intradermal melanocytic proliferation associated with epidermal hyperplasia (hematoxylin and eosin stain [H&E], original magnification 50×). (**B**): The lesion is composed of spindle-shaped and epithelioid cells arranged in nests within a dense collagenous stroma (H&E, original magnification 100×). (**C**): The cells display eosinophilic cytoplasm and ovoid-to-round nuclei with conspicuous nucleoli (H&E, original magnification 200×). (**D**): Immunohistochemical staining for ROS1 shows uniform staining throughout the lesion (ΙHC, original magnification 50×).

*ROS1*-fused tumors represent approximately 10% of all Spitz lesions [52]. Common fusion partners include *TPM3*, *PPFIBP1*, *MYH9*, *CAPRINI1,* and *MYO5A* [8], while less frequent fusion partners include *LIMA1* and *LRRFIP2* [47]. It seems that most *ROS1*-fused tumors do not show distinct and reproducible histopathologic findings [50,51] (Figure 7 and Figure 8).

*MAP3K8* fusions or truncating mutations can be seen in up to one-third of SMs [63], displaying epithelioid morphology, evident cytologic atypia, ulceration, desmoplasia, and a more aggressive biological behavior with frequent spread to lymph nodes [8]. Common fusion partners include *CUBN*, *DIP2C*, *PRKACB*, *STX7*, *SPECC1*, *SVIL,* and *UBL3* [63].

Finally, *MET* or *RET* fusions are rare, detectable in only 0.5–3% of Spitz tumors [8], and their importance lies mostly in the potential for targeted treatment with tyrosine kinase inhibitors [45,64].

### 3.3. Other Molecular Alterations 

*MAP2K1* mutations can be observed in the whole spectrum of Spitz tumors, ranging from Spitz nevi (SN) to Spitz melanoma (SM) [65,66], while *MAP2K1*-activating in-frame deletions have been detected in Spitz melanocytomas with dense pigmentation and mostly indolent clinical course [65]. In addition, *MAP2K1*-activating mutations have been detected in a subset of melanocytic tumors that closely resemble true Spitz neoplasms, showcasing morphologic features such as Kamino bodies and epidermal hyperplasia. Compared to true Spitz neoplasms, these tumors typically affect older individuals and demonstrate a higher propensity for pagetoid spread while displaying lower levels of mitotic activity [67].

Additional cytogenetic findings linked to aggressive clinical behavior include 6q23 deletions in Spitz melanocytomas correlated with expansile nodular architecture, focal ulceration, minimal pagetoid spread, and the development of sentinel lymph node metastases [68], as well as 9p21 deletions, particularly in pediatric Spitz lesions [69,70,71].

*TERT* promoter mutations, although not very common, are a hallmark of the aggressive clinical behavior of Spitz tumors [51,72]. In the study by Lee et al. on 56 patients with Spitz tumors, all 4 patients who died of metastatic disease at the end of the follow-up period had *TERT* promoter mutations. The initial diagnoses were SM in one patient, Spitz melanocytoma in two patients, and SN in one patient. Over the course of disease, the two patients with Spitz melanocytoma and the one patient with SN were reclassified as having SM after being diagnosed with nodal or widespread metastatic disease. Not surprisingly, *TERT* promoter mutations proved to be a statistically significant predictor of hematogenous spread [72]. An additional link between telomere physiology and spitzoid morphologic features is the presence of spitzoid characteristics seen in melanomas arising in patients with germline mutations in *POT1*, a gene involved in telomere maintenance [73]. 

It comes as no surprise that the mRNA expression profile differs among different Spitz lesions, with SN and SM displaying differential expression of *NRAS, NF1, BMP2, EIF2B4, IFNA17,* and *FZD9* [74], and SN showing upregulated immunomodulatory, inflammatory, extracellular matrix interactions, angiogenesis-associated processes relative to common nevi [75]. The microRNA expression profile of Spitz lesions is diverse as well, highlighting the existing differences in their epigenetic status. More specifically, Spitz melanocytomas show upregulation of miR-451a and downregulation of mir-155-5p relative to SM, as well upregulation of miR-21-5p, miR-34a-5p, miR-451a, miR-1283, and miR-1260a relative to SN [76]. Conversely, SN upregulates expression of miR-21-5p and miR-363-3p relative to common nevus [76]. Finally, disrupted epigenetic regulation seems to give rise to Spitz lesions with recurrent mutations in the *KMT* gene family, encoding parts of the methyltransferase complex which is necessary for histone modification [27]. 

A certain degree of interaction between genetic and hormonal factors is very likely to be included in the molecular pathogenesis of Spitz lesions. The slight female predominance seen in Spitz lesions is suggestive of a possible pathogenetic hormonal effect [16]. In addition, patients with thyroid hormone receptor alpha (RTHα) germline mutations are more likely to develop nevi [77].

#### Immunohistochemistry as a Surrogate of Molecular Testing

Mutant-protein specific antibodies can be of great clinical utility. These include BRAF V600E, as well as NRAS Q61R [78]. In fact, the initial step in the immunohistochemical assessment of spitzoid lesions typically involves testing for BRAF V600E and possibly NRAS Q61R, which are expected to be negative in Spitz tumors. Nevertheless, the immunohistochemical expression of NRAS Q61R can be observed in Spitz tumors with the *HRAS* Q61R mutation, reflecting cross-reactivity between different RAS proteins [79].

In addition, the use of a set of immunostains for p16, Ki-67, and HMB45 is frequently recommended for spitzoid lesions [39,80], followed by FISH with probes for 6p25, 8q24, 11q13, centromere 9 and 9p21, and/or array-based CGH for discrepant cases [39,80]. Assessing each of p16, Ki-67, and HMB45 individually can lead to pitfalls, since Ki-67 can be slightly elevated in pediatric tumors and p16 can be completely negative in Spitz melanocytomas due to biallelic loss of 9p21 [51]. HMB45 can occasionally be negative in Spitz melanocytoma or Spitz melanoma (SM) [81]. Several other immunohistochemical markers have been proposed as useful in the differential diagnosis of spitzoid tumors, including cyclin D1, cyclin A, bcl2, BAX, survivin, cKIT, Rb, WT1, COX2, and galectin-3, but results and interpretations have been conflicting, limiting their clinical utility [82]. 

Applying immunohistochemistry to identifying Spitz tumors associated with fusions has recently been gaining ground, particularly regarding ALK and pan-NTRK, since tumors with either *ALK* or *NTRK1/2/3* fusions typically show diffuse and strong immunoreactivity with the corresponding antibodies [48,52,57], while antibodies for ROS1 can show weak reactivity and imperfect sensitivity [52]. Non-specific background staining can render the interpretation of NTRK immunostaining challenging. However, the data suggest that all tumors with a negative or weak immunohistochemical reaction are negative for *NTRK1/2/3* fusions by molecular testing, indicating a negative predictive value of 100% [57]. A pitfall mentioned earlier is that the widely used ALK1 antibody clone exhibits lower sensitivity compared to the 5A4 or D5F3 antibodies in detecting ALK expression in *ALK*-fused tumors [54]. 

The use of immunohistochemistry for detecting the expression of the preferentially expressed in melanoma antigen (PRAME), a tumor-associated antigen expressed, among others, by melanoma, myxoid liposarcoma, and synovial sarcoma [83], has shown some clinical value in the differential diagnosis of spitzoid lesions. Specifically, *PRAME* gene expression is significantly increased in melanomas related to SN [84] and immunoreactivity is subsequently seen more frequently in SM [85]. However, PRAME immunohistochemical expression does not correlate well with underlying chromosomal alterations and its specificity is imperfect [38].

## 4. Concluding Remarks

After a 75-year journey in the research of Spitz lesions and well into the era of genomic medicine, the clinical challenge of classifying difficult spitzoid lesions persists. Facing the future, there is hope that novel developments in artificial intelligence (AI) and deep learning may evolve into useful clinical tools, although substantial progress is still needed. Early efforts to utilize AI algorithms have faced challenges due to the “a priori” diagnostic criteria applied by pathologists in defining which lesions will be diagnosed as Spitz nevi (SN), Spitz melanocytomas, or Spitz melanomas (SMs) [86]. Although a recently published model for classifying Spitz lesions based on whole slide imaging (WSI) showed promising results [87], diagnosing Spitz lesions with an integrated morphologic and genomic approach is likely to remain the standard of practice in the near future. Ideally, challenging cases with ambiguous morphology should be sequenced and histologic findings should be reappraised under the light of sequencing results in order to reach a definitive classification [88]. In conclusion, we believe that the role of practicing pathologists in integrating clinicopathologic and genomic data to establish an accurate and timely diagnosis of Spitz lesions will remain of paramount importance for proper patient management [86].
